# Supplementary material for: Natural scene sampling reveals reliable coarse-scale orientation tuning in human V1
Source: Nat Commun. 2022 Oct 29;13:6469. doi: 10.1038/s41467-022-34134-7 (PMC9617970; doi:10.1038/s41467-022-34134-7)
Supplement: Supplementary file 1 — Supplementary Information [file 41467_2022_34134_MOESM1_ESM.pdf]

Supplementary Materials for

Natural scene sampling reveals reliable coarse-scale orientation tuning in human V1

Zvi N. Roth*, Kendrick Kay, Elisha P. Merriam,

*Corresponding author. Email: zvi.roth@nih.gov

This PDF file includes:

Figs. S1 to S4

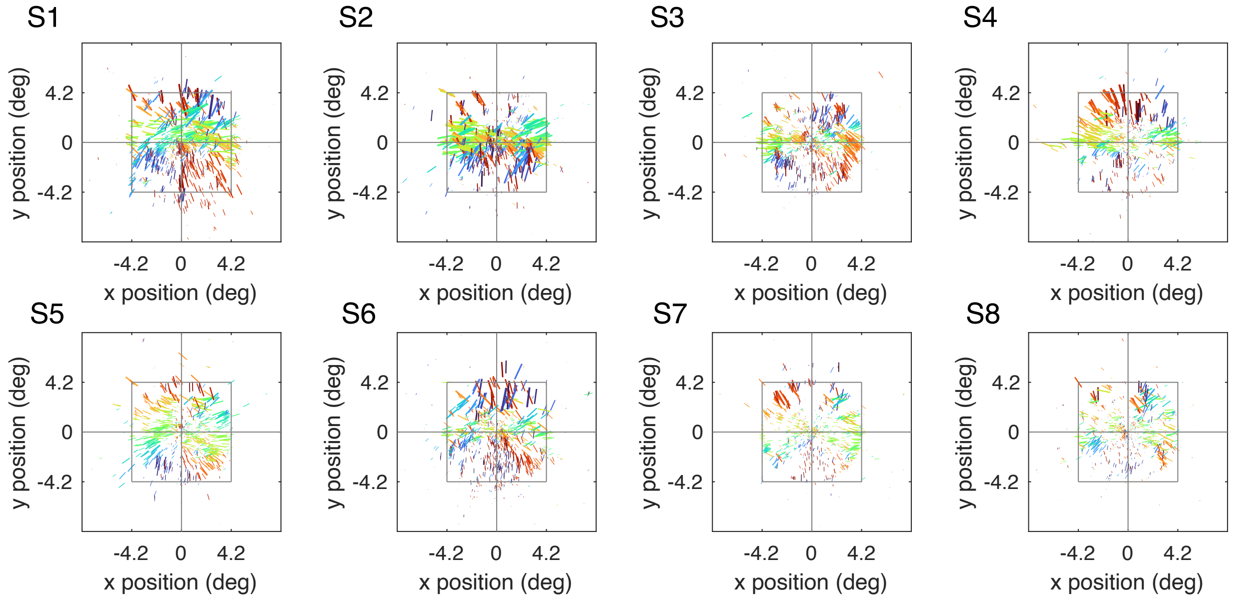

Supplemental Fig S1. Orientation maps are consistent across participants. Same color legend and scaling as Fig 3A. Orientation preferences plotted in visual space. Each line represents a single voxel and is positioned at the voxel's pRF center. Hue and orientation of the line indicate preferred orientation. Line length, width, and scale reflect the amount of variance (R^2) explained by the constrained model. Solid square at +/- 4.2 deg indicates the size of the natural-scene stimuli.

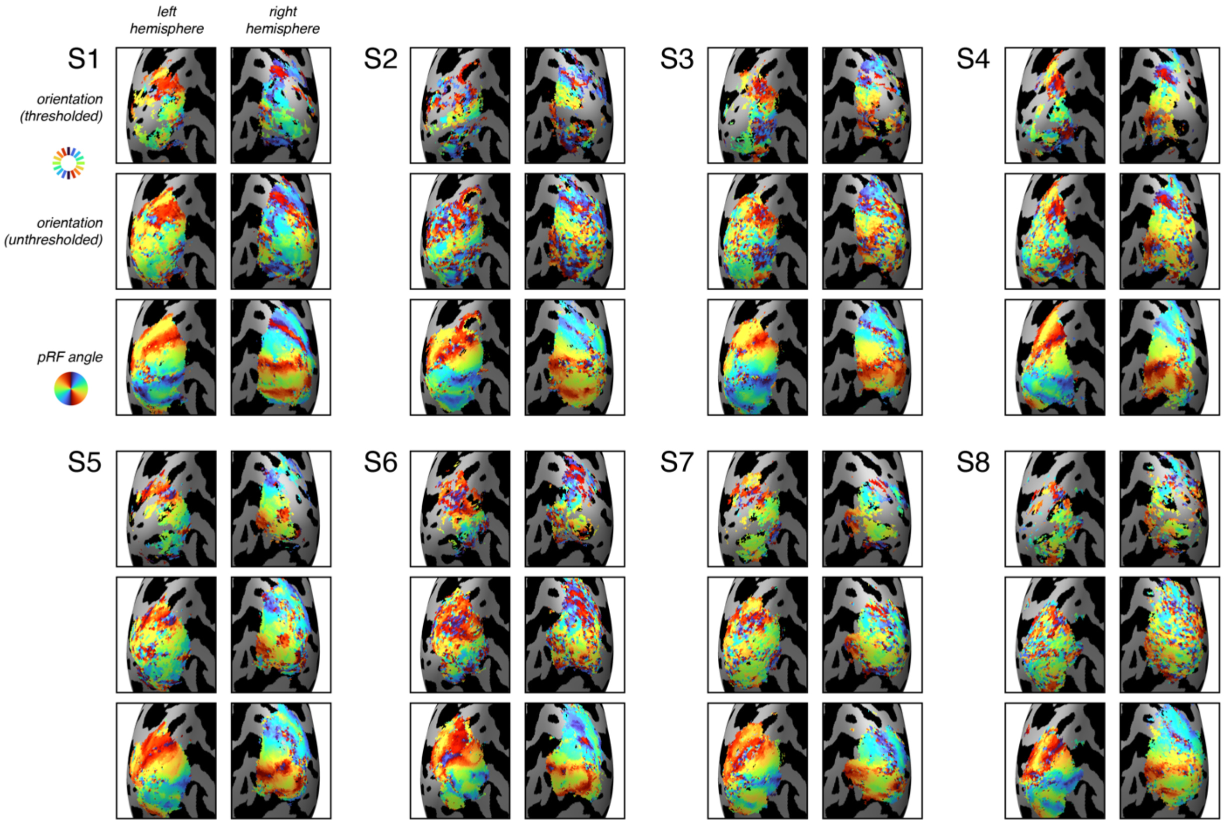

Supplemental Fig S2. **Orientation surface maps are consistent across individuals**. Same color legend as Fig 3B. Individual subject orientation maps (top, thresholded; middle, unthresholded) and pRF polar angle maps (bottom) overlaid on left and right inflated 'fsaverage' surfaces. For angle map and unthresholded orientation map, all vertices in V1, V2, V3, and V4 are plotted. For thresholded orientation map only vertices with the top 50% full model R^2 are plotted.

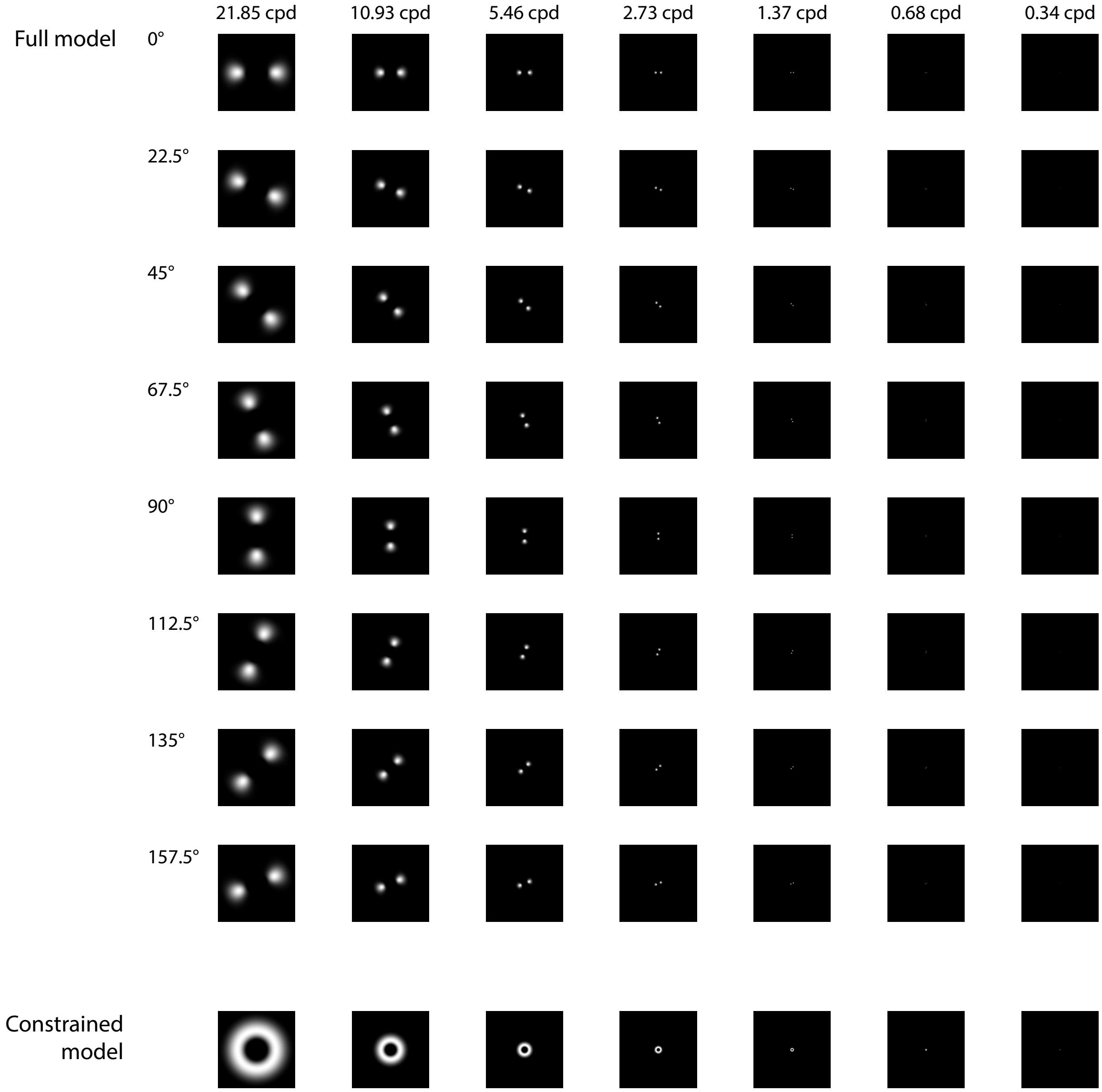

Supplemental Fig S3. Full model and constrained model filters in Fourier space. The full model allows unequal contributions from orientation-selective filters, and is therefore sensitive to orientation-selectivity beyond the effects of stimulus vignetting. The constrained model pools equally across orientation-selective filters, and therefore has no orientation selectivity, but due to spatial frequency tuning it accounts for effects of stimulus vignetting.

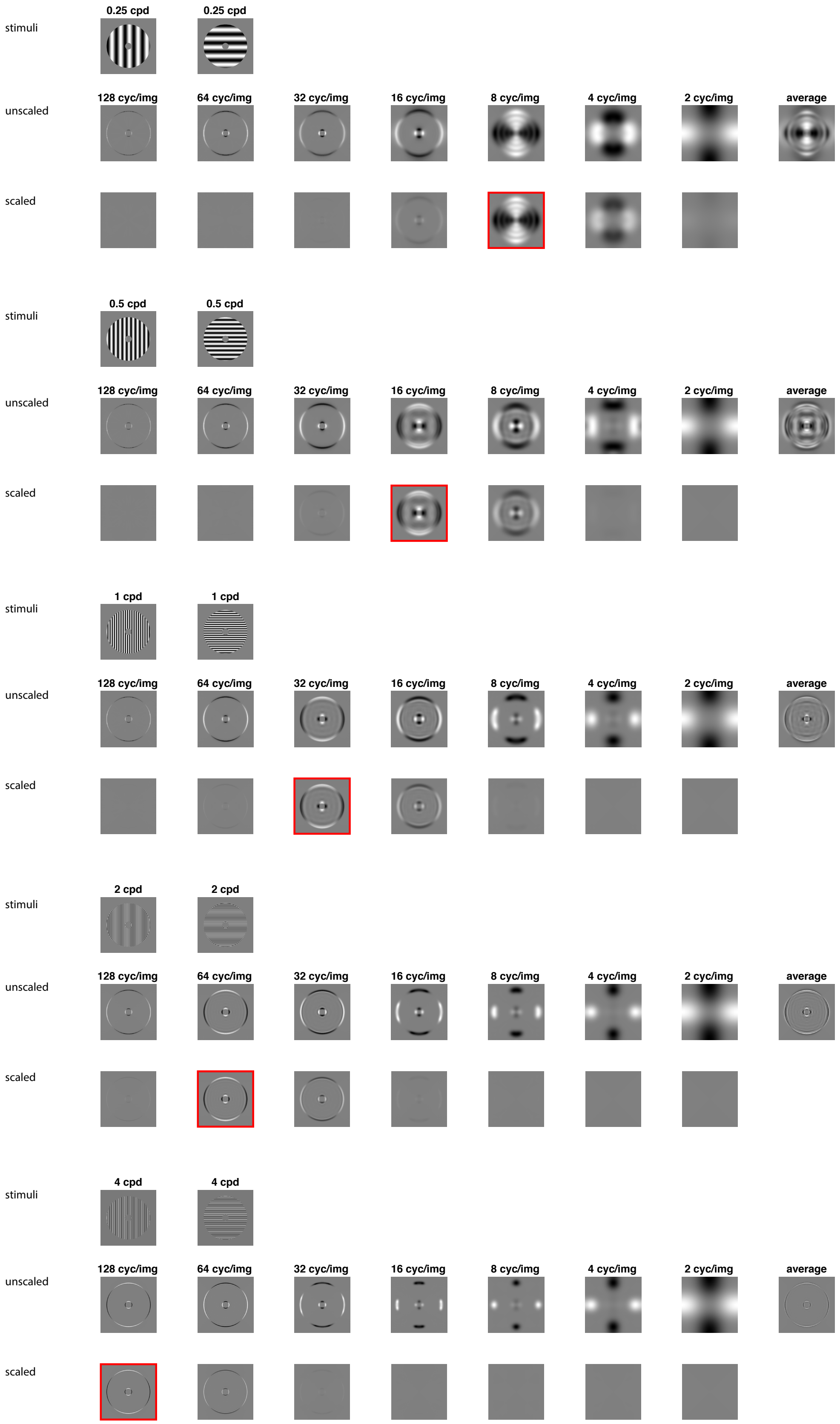
stimuli
0.25 cpd
0.25 cpd
unscaled
128 cyc/img
64 cyc/img
32 cyc/img
16 cyc/img
8 cyc/img
4 cyc/img
2 cyc/img
average
scaled
stimuli
0.5 cpd
0.5 cpd
unscaled
128 cyc/img
64 cyc/img
32 cyc/img
16 cyc/img
8 cyc/img
4 cyc/img
2 cyc/img
average
scaled
stimuli
1 cpd
1 cpd
unscaled
128 cyc/img
64 cyc/img
32 cyc/img
16 cyc/img
8 cyc/img
4 cyc/img
2 cyc/img
average
scaled
stimuli
2 cpd
2 cpd
unscaled
128 cyc/img
64 cyc/img
32 cyc/img
16 cyc/img
8 cyc/img
4 cyc/img
2 cyc/img
average
scaled
stimuli
4 cpd
4 cpd
unscaled
128 cyc/img
64 cyc/img
32 cyc/img
16 cyc/img
8 cyc/img
4 cyc/img
2 cyc/img
average
scaled

Supplemental Fig S4. Response of constrained model filters to circular apertured gratings at various spatial frequencies. Responses to vertical orientation minus responses to horizontal orientation are shown for all 7 filters, for 5 different grating spatial frequencies. Stimulus is 512 × 512 pixels, here simulated to span 10 × 10 degrees of visual angle. For each spatial frequency, responses are averaged across 8 different phases. Unscaled responses (middle row for each spatial frequency) shows responses scaled from the minimum (black) to maximum (white) for each individual filter. This enables one to see how the spatial patterns change across the different filters, but doesn't convey a sense of the relative magnitude. In the bottom row of each spatial frequency the gray scale for all panels ranges from the minimum to maximum across all filters ('scaled'). The filter response corresponding to the stimulus frequency has the largest response, ranging from black to white, but other filters have smaller responses, which are therefore washed out and almost invisible. The model filter that corresponds to the grating spatial frequency (marked with a red box) exhibits a radial bias, while the adjacent filters show an opposite effect. However, when scaling all filters to the same maximal value it becomes apparent that the effects in adjacent filters are much weaker than that of the filter with the maximal response. Therefore, as long as the spatial frequency of the stimulus is matched to the preferred spatial frequency tuning of a local population of neurons in a voxel, and assuming a circular aperture, vignetting should result in a radial bias.
